# Heterogeneous Urban Exposures and Prevalent Hypertension in the Helsinki Capital Region, Finland

**DOI:** 10.3390/ijerph18031196

**Published:** 2021-01-29

**Authors:** Enembe O. Okokon, Tarja Yli-Tuomi, Taina Siponen, Pekka Tiittanen, Anu W. Turunen, Leena Kangas, Ari Karppinen, Jaakko Kukkonen, Timo Lanki

**Affiliations:** 1 Environmental Health Unit, Department of Health Security, Finnish Institute for Health and Welfare, Neulaniementie 4, P.O. Box 95, FI-70701 Kuopio, Finland; tarja.yli-tuomi@thl.fi (T.Y.-T.); taina.siponen@thl.fi (T.S.); pekka.tiittanen@thl.fi (P.T.); anu.turunen@thl.fi (A.W.T.); timo.lanki@thl.fi (T.L.); 2 Department of Community Medicine, Faculty of Medicine, University of Calabar, Calabar PMB 1115, Nigeria; 3 Department of Atmospheric Composition Research, Finnish Meteorological Institute, Erik Palménin Aukio 1, FI-00560 Helsinki, Finland; leena.kangas@fmi.fi (L.K.); Ari.Karppinen@fmi.fi (A.K.); Jaakko.Kukkonen@fmi.fi (J.K.); 4 Department of Environmental and Life Sciences, University of Eastern Finland, 70211 Kuopio, Finland

**Keywords:** wood smoke PM_2.5_, road-traffic PM_2.5_, road-traffic noise, green areas, hypertension

## Abstract

Urban dwellers are simultaneously exposed to several environmental health risk factors. This study aimed to examine the relationship between long-term exposure to fine particulate matter (PM_2.5_, diameter < 2.5 µm) of residential-wood-burning and road-traffic origin, road-traffic noise, green space around participants’ homes, and hypertension. In 2015 and 2016, we conducted a survey of residents of the Helsinki Capital Region to determine their perceptions of environmental quality and safety, lifestyles, and health statuses. Recent antihypertensive medication was used as an indicator of current hypertensive illness. Individual-level exposure was estimated by linking residential coordinates with modelled outdoor levels of wood-smoke- and traffic-related PM_2.5_, road-traffic noise, and coverage of natural spaces. Relationships between exposure and hypertension were modelled using multi-exposure and single-exposure binary logistic regression while taking smooth functions into account. Twenty-eight percent of the participants were current users of antihypertensive medication. The odds ratios (95% confidence interval) for antihypertensive use were 1.12 (0.78–1.57); 0.97 (0.76–1.26); 0.98 (0.93–1.04) and 0.99 (0.94–1.04) for wood-smoke PM_2.5_, road-traffic PM_2.5_, road-traffic noise, and coverage of green space, respectively. We found no evidence of an effect of the investigated urban exposures on prevalent hypertension in the Helsinki Capital Region.

## 1. Introduction

Urban living is associated with some health risks which are related to situational factors including air pollution and noise exposure [1]. Key environmental attributes which dominate urban exposure have shown consistent associations with poor health outcomes [2]. The most important environmental factor is particulate air pollution [3]. Particulate matter (PM) refers to a continually changing mixture of inorganic and organic solid and liquid particles in aerosol state, and it is constantly present in the atmosphere [4].

A large body of research shows a causal relationship between PM_2.5_ and cardiovascular diseases [5,6,7]. Empirical evidence also relates fine particles with a higher risk of hypertensive disease following both short-term [8,9] and long-term [10,11,12] exposure. Notwithstanding, this relationship is sometimes less certain [13,14]. Although a large proportion of locally sourced urban PM_2.5_ is generated by road-traffic in most developed countries, domestic energy production may significantly contribute to outdoor levels. For example, in Finland, domestic wood burning is currently the most important source of PM_2.5_ emissions [15]. Incidentally, vehicular emissions exceeded domestic wood burning emissions in the Helsinki Capital Region in the years preceding the middle of the first decade of the 21st century [16]. The adverse health effects of domestic wood burning are mostly attributed to fine particles, which are released during the combustion process [17]. Domestic wood burning is more widespread in high-income countries, with growing preference because it supplements the energy budget for domestic heating or is put to recreational use [18,19].

When inefficient combustion appliances are used in poorly ventilated spaces, domestic wood burning can lead to considerable indoor PM_2.5_ exposure [17]. A recent Finnish study showed that relatively short-lasting residential wood burning increased occupants’ daily exposure to wood smoke [20]. Indoor-generated wood smoke can combine with outdoor wood smoke and traffic-related PM_2.5_ infiltrating into the indoor space to cause long-term exposure in urban dwellings. Thus, co-exposure is typical if both fine particles from wood stoves and road-traffic are present. Notably, the contributions of traffic and wood smoke to urban PM_2.5_ levels vary by time and place [21]. Wood smoke can dominate overall ambient PM_2.5_ levels in some cities [15,17,21], especially during colder seasons when residential indoor heating is unavoidable. Furthermore, recreational use of wood heating in sauna stoves may have a high impact on local PM_2.5_ concentration [15,16]. Road traffic-related PM_2.5_ exposure assumes higher significance where residential dwellings are proximate to major roads.

Road-traffic is a prominent source of urban air pollution [22,23], and also the most common source of environmental noise [23,24]. The 2011 Environmental Burden of Disease report estimates that in Europe, 903,000, 654,000 and 61,000 disability-adjusted life years (DALYs), respectively, are lost annually from noise-induced sleep disturbance and annoyance, and noise-attributable coronary heart disease [24]. Fine particles and noise can affect blood pressure via distinct and shared physiological pathways: PM_2.5_ induces a pro-inflammatory response [5,25,26,27] which could result in the loss of endothelial elasticity and vascular stiffness [28]. Fine particles can also induce an autonomic imbalance in hemodynamics [29]. Noise, on the other hand, stimulates stress reaction via the endocrine and autonomic pathways [30], leading to an increased blood pressure. The shared autonomic pathway raises the possibility of an additive or synergistic effect, but there is insufficient evidence to suggest an effect modification between these pollutants [31].

While some environmental exposures increase the risks of harmful effects, others have been suggested to yield salutary effects on human health. Evidence tends to suggest that access to green areas may offer a counterweight to some urban health risks [32,33,34]. For example, green areas have been observed to have restorative effects on city dwellers [35,36]. Proximity to a green area seemingly improves mental health [37,38] and vitality [39] by relieving stress [40]. Green space also seems to have some beneficial effects on cardiovascular health [39,41], but existing research on the effects of green space on blood pressure present mixed findings [42].

An experimental study by Lanki and colleagues revealed that blood pressure decreased when women sat in the forest to view the surrounding scenery [43]. Part of this effect was attributable to the green environment itself rather than lower air pollution and noise levels in the forest. A systematic review reported no relationship between access to green space and arterial blood pressure [31], but that finding has been countered by a more recent systematic review [44]. Moreover, some other reports have shown an association between higher residential greenness and lower blood pressure in children [45], as well as self-reported park use and a lower prevalence of hypertension in adults [41]. The proposed mechanisms for the observed benefits of green areas include enhanced opportunities for exercise, social interaction, solitude, and contact with natural elements which trigger positive emotions [34,35]. Green spaces tend to experience lower volumes of road traffic, and are, therefore, subject to lower air pollution and noise levels.

Owing to the broad array of urban exposures and the paucity of studies which simultaneously investigate these exposures relative to hypertensive risk, we hypothesize that: (1) residential wood burning, road traffic-related air pollution, and noise are risk factors for hypertension; (2) proximity to green space reduces the risk of hypertension. Our study, therefore, aims to assess the risk of hypertension among urban dwellers of Helsinki, Espoo, and Vantaa in relation to local levels of wood-smoke PM_2.5_, road-traffic PM_2.5_, road-traffic noise, and green space coverage near their homes.

## 2. Materials and Methods

We developed this study using data from the Helsinki Capital Region Environmental Health Survey. The Helsinki Capital Region, which consists of Helsinki, Vantaa, and Espoo, is the most densely populated area in Finland. It provides a large enough population for sampling in an area small enough to be is feasible for high-resolution modelling of environmental exposures. In addition, there is an exceptionally detailed, high spatial resolution multidecadal dataset which includes pollution concentrations for a period of 35 years in the Helsinki Metropolitan Area. This dataset has been described in detail by Kukkonen et al. [16]. This dataset also includes also source category-specific concentrations. Such datasets are very rarely available around the world for health assessment studies.

This Helsinki Capital Region Environmental Health Survey was conducted in the summer months of 2015 and 2016. It is described in detail elsewhere [46], but briefly, the survey was conducted to determine the residents’ perceptions of their personal exposures and risks in relation to an array of environmental factors. The survey also elicited outcome and covariate data for environmental epidemiology studies. The survey involved the completion of a self-administered questionnaire which was presented in electronic format and as a paper copy. The residential dwellings of the respondents were linked with air pollution maps, noise maps, and GIS data on natural spaces.

### 2.1. Exposure Assessment

#### 2.1.1. Subjective Exposure Assessment

Subjective wood-smoke exposure was elicited using the question, “On average, how often do you burn wood at home during the cold season (October to April)?” Respondents had the following options to choose from: “Less than once a month/1–3 times a month/1–2 times a week/3–4 times a week/5 times a week or more often”.

#### 2.1.2. Objective Exposure Assessment

##### Estimation of Woodsmoke PM_2.5_

The Finnish Meteorological Institute has modelled the concentrations of PM_2.5_ globally, in Europe, and in the Helsinki Capital Region for a period of 35 years, from 1980 to 2014 [16]. In the Helsinki Capital Region, emissions which originate from domestic wood combustion are uniformly distributed in area sources of dimensions 100 × 100 m^2^. The corresponding concentrations were evaluated using the Urban Dispersion Model of the Finnish Meteorological Institute (UDM-FMI) on a horizontal resolution of 100 × 100 m^2^ [16]. In the present study, the PM_2.5_ concentrations which were assigned to participants’ homes were evaluated as the wood-smoke concentrations at the nearest outdoor modelling point to each home. An average value, which spanned the years 2010 to 2014, was used as the residential exposure level.

##### Estimation of Road-Traffic PM_2.5_

Estimates of traffic-sourced PM_2.5_ concentrations in the Helsinki Capital Region were based on emission and dispersion modelling which used vehicular exhaust emission factors from 2014 and the road network (CAR-FMI) dispersion model [47]. The distances between the receptor points varied from 25 m near the roads to 200 m in rural areas. In the present study, the PM_2.5_ concentrations were assigned to participants’ homes as the estimated PM_2.5_ concentrations at the nearest outdoor modelling point to each home. The highest annual average values for the years from 2010 to 2014 were used as the exposure estimates.

##### Estimation of Road-Traffic Noise

The noise exposure model took into account the direction of windows in residential dwellings. Façade noise levels from road-traffic were calculated by a Finnish consulting company, Sito, in accordance with the EU Environmental Noise Directive 2002/49/EC50 using input data for the year 2016. The Common Noise Assessment Methods in Europe (CNOSSOS-EU) method [48] was used for major highways, main streets, and collector streets within areas. All L_den_ (day-evening-night equivalent levels) which equate to or exceed 30 dB on façade points within 20 m of residential address coordinates were selected for each home. If the windows of a residential dwelling faced the street, implying a higher noise exposure, the highest L_den_ was assigned as the noise exposure to that dwelling. Conversely, if the windows faced the yard, the lowest L_den_ was assigned as the noise exposure. In situations where windows faced both the street and the yard, an average of the highest and lowest noise levels was used.

##### Estimation of Nature Spaces

The Urban Atlas 2012 (European Environment Agency, Copenhagen, Denmark, 2012) was used to determine the percent coverage of either green or blue areas within buffer zones of 300 m and 1 km around each home. The ArcMap 10.5 (Esri, Redlands, CA, USA) was used to calculate the surface areas of green and blue spaces within the buffers. Arable land, pastures, forests, green urban areas, herbaceous vegetation associations, and open spaces with little or no vegetation were designated as green areas, while sea, lakes, rivers, and wetlands were designated as blue areas.

### 2.2. Outcome Assessment

The outcome of interest was a current history of hypertension. This was determined in two ways: firstly, we derived a proxy indicator of hypertension to ascertain recent or current use of antihypertensive medication. This was achieved by asking the generic question: “*When did you last take the following medication?*” Respondents could then choose one of the following options (from a list of medication broadly categorized according to the medical conditions for which the medicines are given, including hypertension): “*during the past week/1–4 weeks ago/1–12 months ago/over a year ago/never*”. Only persons who used antihypertensive medication in the preceding week were considered users. All others were categorized as non-users. Alternatively, we categorized respondents as self-reported hypertensives if they reported that they had been diagnosed as having hypertension, or received treatment for hypertension from a physician within the past 12 months. Self-reported non-hypertensives were defined as those who were neither diagnosed nor treated.

### 2.3. Covariates

The initial choice of covariates was based on a priori knowledge of select risk factors, and these were consistent with covariates which Fuks and Weinmayr [49] used in the ESCAPE study on arterial blood pressure (BP) and air pollution. This set of covariates consisted of sex, age, marital status (single/married or cohabiting/widowed), educational status (basic education (comprehensive school)/vocational education/general upper secondary education/higher education (universities and universities of applied sciences)) [50], employment status (full time/part-time or student/retired or homemaker/unemployed) and annual household income before tax (≤EUR 30,000/EUR 30,001–50,000/EUR 50,001–70,000/>EUR 70,000). In addition, it included BMI (≤18.5/>18.5–25/>25–30/>30 (kg/m^2^)), leisure-time exercise (never/1–2 times per week/3–4 times per week/>4 times per week), smoking status (yes/no), passive smoking (yes/no), alcohol consumption, area-level mean income and area level unemployment rates. Alcohol consumption was based on an aggregate of the number of bottles or glasses or measures of the pertinent alcoholic beverage consumed weekly (≤3 (mild)/>3–4 (moderate)/>4–7 (severe)/>7 (extreme)). To accommodate a prevalent sociocultural attribute of our study population, the use of a summer retreat home was added to the list of covariates. Ownership and use of a summer retreat home is common in the Finnish population. These homes are often located away from the city, in the woods and/or beside lakes. The summer cottage offers some escape from the pressures of city life, opportunities for relaxation, restorative benefits, and creative physical activity. Times spent in summer homes can modify the effects of urban exposure.

### 2.4. Statistical Analysis

We compared the distribution of the covariates between hypertensives and non-hypertensives using Pearson’s chi square for categorical data and Student’s *t*-test for parametric data. We determined the correlation between exposures using Pearson’s product momentum correlation coefficient. We used the binary logistic regression to examine the prevalence of the use of antihypertensive medication as explained by exposures comprising wood-smoke PM_2.5_, road-traffic PM_2.5_, road-traffic noise, and coverage of green areas around home. All exposures were simultaneously adjusted for in multi-exposure models, but we also performed single-exposure analyses. In the crude models, we controlled only for age and sex. The main models accommodated elements of the crude model and also household income, mean area-level income, employment status, alcohol consumption, active smoking, passive smoking, BMI, and physical exercise. These models were checked for linearity by visual inspection of regression plots and by using the ‘car’ package, in the R computing environment. Only age and area-level mean income showed non-linear associations with the outcome. Therefore, a smooth term was added to age in all statistical models and to area-level income in the pertinent models using the penalized spline. The penalized spline function models non-linear relationships with an algorithm which automatically assigns knots to produce the model best fit. Thus, these models are insensitive to the placement of knots. We specified three (3) degrees of freedom to the spline function after preliminary examination.

We further explored antihypertensive medication use relative to the environmental exposures using several sensitivity models. In the sensitivity model 1, access to blue space, use of a summer home, and marital status were added to the main model. Educational status and area-level unemployment rate substituted for household income and area-level mean income. The objective was to see if using other indicators of socioeconomic status affected outcome estimates. In the sensitivity model 2, wood burning frequency was added to the main model. In the sensitivity model 3, percent coverage of green space within a distance of 300 m replaced percent coverage of green space within a 1 km buffer.

We also tested effect modification by introducing pairwise interaction terms between either of these variables: coverage of green areas, road-traffic noise, and road-traffic PM_2.5_. We tested interactions involving noise measured in either the decibel or Pascal scales since road-traffic noise better correlates with road-traffic PM_2.5_ in the linear Pascal scale. We also tested effect modification between the use of a summer cottage and road-traffic pollutants. We used the Wald chi square to assess the statistical significance of interaction models. The odds ratios for road-traffic noise were estimated for every 5 dB increase, while odds ratios for green space coverage were scaled by 10. To increase statistical power, the BMI category <18.5 kg/m^2^ was merged with the category 18.5–25 kg/m^2^. All statistical tests were conducted at the 95% confidence level and odds ratios were used to represent relative risks. All statistical analyses were conducted using R version 3.3.3 [51].

## 3. Results

Altogether, we had complete records for 5441 study participants. A higher proportion (57%) of study participants were female. The mean age was 56.6 ± 16.4 years and approximately 41% of the study participants were aged 60 years and above (Table 1). Approximately 13% of all participants practiced any residential wood burning (Table 1). Twenty-six percent of participants used antihypertensives within the past week, while 27% had been diagnosed or treated for hypertension by a physician. Those using antihypertensives were generally older, had higher BMI and lower education status than non-users. Antihypertensive users were more often retired from employment or homemakers.

### 3.1. Summaries and Correlation of Exposure Data

The summaries of environmental exposures and the matrix from the Spearman’s correlation coefficients are provided in Table 2. The concentrations of PM_2.5_ originating from the two local sources were low. The correlation coefficients generally indicated weak but statistically significant correlations between exposure variables, except between road traffic-related PM_2.5_ and road-traffic noise, where a moderate positive correlation (r = 0.51) was observed. Green space correlated with road-traffic particulates and road-traffic noise negatively, but showed a positive correlation with wood smoke PM_2.5_.

### 3.2. Associations between Environmental Exposures and Hypertension

The statistical models produced raised effect estimates for wood smoke PM_2.5_, but there were no statistically significant associations with antihypertensive use in the main model or single-exposure model. There was no association between antihypertensive medication and either traffic-derived particulates or traffic noise in the crude models, main models or single pollutant models (Table 3). No effect modification was observed between road-traffic PM_2.5_, road-traffic noise and coverage of green space (result not shown). Estimates from sensitivity models were largely similar to the main model. The weekly frequency of wood burning showed no relationship with the prevalence of antihypertensive medication (Table 4). Using self-reported physician diagnosis or treatment for hypertension as an indicator of recent hypertensive state did not reveal any association between environmental exposures and the use of antihypertensive medication (Table 5).

## 4. Discussion

We examined the relationships between fine particles from traffic and wood combustion, traffic noise, and green areas, and indicators of hypertension. In the study area, the levels of particulate air pollution were low, but the coverage of green areas were relatively high. We did not find associations between the environmental exposures and the use of antihypertensive medication or self-reported physician-diagnosed hypertension. Exploring relationships by applying different model specifications did not change the results.

### 4.1. Woodsmoke Particulates and Antihypertensive Use

We did not find a clear relationship between woodsmoke PM_2.5_ and antihypertensive medication. Some investigators have found the possible effects of wood smoke on excess hypertensive risk. For example, one investigation [52] suggested that a reduction in the exposure of Guatemalan women to wood smoke reduced their risk of hypertension. The experimental nature of the Guatemalan study, the repeated measurement design, and the higher exposure levels gave that study higher statistical power to detect relationships compared, to our study. However, it should also be noted that identical effects are not to be expected, because in the Guatemalan study, the nature of combustion was different, and much of the exposure can be assumed to have taken place right after emission, in which case the study participants were exposed to more reactive chemical species. A related study [53] found that using a high-efficiency particulate air (HEPA) to reduce the amount of inhaled wood smoke PM_2.5_ led to an increase in vascular hyperemia—an index of improved vascular endothelial action—in study participants. This experimental study differs from ours both in design and the use of a subclinical index as a proxy of improved blood pressure. In contrast with the studies discussed above, Clark et al. [54] found that wood smoke PM_2.5_ was neither associated with systolic or diastolic blood pressure in Nicaraguan women.

The toxicity of wood smoke depends on the physicochemical properties which are determined by the efficiency of the combustion appliance in use as well as the conditions of combustion [55]. Wood smoke particles seem to harm cardiovascular health by a molecular mechanism which is congruent with that of traffic-sourced particles [55]. The absence of a clear hypertensive risk in our study may be due to the low exposure to wood smoke PM_2.5_ and the low exposure contrasts in the population. Within most urban locations in the Helsinki Capital Region, the long-range transport contributions to overall PM_2.5_ levels are higher than the contributions from local sources [16]. Previous studies have shown that the most health-relevant air pollutant concentrations in the Helsinki region were lower than the corresponding values in major central and southern European cities [56,57].

Some studies do not use antihypertensive medication as an endpoint in itself, but tend to adjust for medication use in models which examine measured BP levels in association with particulate exposure [58,59,60]. Medication for hypertension is initiated when BP cannot be controlled using non-medicinal approaches such as diet modification, exercise, cessation of smoking and alcohol, and weight loss. We acknowledge that using antihypertensive medication as a proxy for hypertension ignores early hypertensives who have not yet commenced medication.

### 4.2. Road-Traffic Particulates and Antihypertensive Use

In this study, antihypertensive use was not associated with road-traffic-sourced PM_2.5_. Our study replicates the findings of Fuks and Weinmayr [49] which show no influence of road-traffic PM_2.5_ on the prevalence of the use of BP lowering medicines. Another study [60] defined hypertension by BP measurement (systolic BP ≥ 140 mm Hg or diastolic BP ≥ 90 mm Hg), self-report of doctor-diagnosed hypertension or use of antihypertensives medication. This study found a 15% change (95% CI: 2.5–28.0%) in the prevalence of hypertension associated with PM_2.5_ in a single pollutant model and an 18% (95% CI: 3.9–36.2%) prevalence in a two-pollutant model adjusted for NO_2_. The working definition of hypertension used by these authors [60] enables a wider capture of persons with the outcome, and would, thus, increase the statistical power of the models. Measurement of BP of study participants likely revealed persons with raised BP who might not yet have been clinically diagnosed. This difference in specification of hypertensive subjects may explain in part the inconsistency of findings from that study and ours. Other studies, which report elevated risks of raised BP with road-traffic air pollution, do not directly compare with ours, as these used different but related endpoints. Some studies have investigated PM_2.5_ effects on BP in persons who were already at high risk due to existing cardiac conditions [9] or the presence of known risk factors [29,61,62,63]. Our study investigated the general adult population with no specific bias toward persons with cardiovascular risks.

### 4.3. Nature Spaces and Antihypertensive Use

Our study did not reveal any attenuating effects of green spaces on the use of antihypertensives. The study sample represented an urban population. There was no difference in the mean green space coverage between users of antihypertensives compared to non-users. This was true at 1 km and 300 m buffers. The majority of the residential dwellings had no access to blue space, while more than half of the remaining had very little blue space coverage. Green spaces offer opportunity for exercise, social interaction, restive solitude and psychological restoration. While this and another cross-sectional study [42] observed no relationship between green space coverage and the prevalence of hypertension, other studies which used the same study design [64,65] found an inverse relationship between green space coverage and hypertension.

### 4.4. Sensitivity Analyses

Our model estimates were robust to all sensitivity analyses. In addition, using physician-diagnosed hypertension as a substitute outcome in multi- and single-exposure models gave fairly similar estimates to results seen with the antihypertensives-user endpoint. It is instructive that questions on the use of antihypertensives and physician diagnosis of hypertension were constructed differently, and scored on different scales. We envisage a considerable overlap between these measures, as suggested by the nearly equivalent proportion of persons who used antihypertensives and those diagnosed or by a physician. Obtaining similar results strengthens our confidence in the study findings.

### 4.5. Strengths and Weaknesses

Studies which investigate simultaneously the effects of air pollution, noise, and green areas are rare in the literature. Exploring the concurrent effects of these exposures on a clinical endpoint expands our understanding of the relative influences of these exposures acting together. In general, studies on the long-term effects of wood smoke exposure are rare. More commonly, investigators tend to model forest fire-related PM_2.5_ and assess the health outcomes on the general population. Assessments involving locally emitted wood smoke particulates are uncommon. Exposure assessment is one of the strengths of this study. The emissions and atmospheric dispersion of PM_2.5_ from the two sources were modeled in high resolution. The estimation of noise exposure, which regards the orientation of windows, minimizes exposure misclassification. Finally, the results from this study have the benefit of a large sample size, and statistical adjustment for a robust set of covariates. These considerations restrain the levels of uncertainty in our estimates.

An apparent weakness in our study is the use of recent antihypertensive medication as proxy for hypertension which omits early hypertensives who have not yet started taking medicines. This introduces some level of misclassification in the determination of the outcome. In addition, we did not elicit the history of antihypertensive use by the specific class of drug. Rather, our question extracted the broad clinical indication for the prescription. There is the remote possibility that study subjects may have misclassified their medication as an antihypertensive when they were actually on some other therapeutic remedy. Contrary to this concern, it has been argued that eliciting blood pressure lowering medication produces less false positives than actual blood pressure measurements [14]; in which case, the strength of association should be higher with the antihypertensive outcome. Finally, the cross-sectional study design is useful in preliminary epidemiologic research, but its key limitation is that it does not take into account the temporality of relationships.

## 5. Conclusions

In conclusion, multiple urban exposures are the norm, but the impact of several environmental exposures simultaneously affecting a given health outcome is still poorly understood. This study was conducted in an urban area with low air pollution levels and relatively high coverage of green areas. In this setting, we found that exposure to source-specific fine particulate air pollution, traffic noise, and urban green areas were unrelated to hypertension. The relationships between these exposures and effect should be simultaneously investigated in the future using longitudinal study designs and several indicators of hypertension as outcome, including measurements of blood pressure.

## Figures and Tables

**Table 1:** Descriptive data of study participants.

	Total N ^a^	%	Not Using Antihypertensives		Used Antihypertensives in Preceding Week	
			n ^b^	%	*n*	%
**Sex**	**5441**					100
females	3095	56.9	2330	57.7	765	54.4
males	2346	43.1	1706	42.3	640	45.6
**Age (years) ^c^**	**5441**	56.6	49.6	15.0	66.3	11.8
**BMI (kg/m^2^)**	**5441**					
14–25	2667	49.0	2252	55.8	415	29.5
25.01–30	1949	35.8	1341	33.2	608	43.3
>30	825	15.2	443	11.0	382	27.2
**Smoker**	**5441**					
no	4572	84.0	3384	83.8	1188	84.6
yes	869	16.0	652	16.2	217	15.4
**Passive smoker**	**5441**					
no	4493	82.6	3374	83.6	1119	79.6
yes	948	17.4	662	16.4	286	20.4
**Employment status**	**5441**					
fulltime	2880	52.9	2490	61.7	390	27.8
part-time/student	436	8.0	373	9.2	63	4.5
retired/homemaker	1868	34.3	971	24.1	897	63.8
unemployed	257	4.7	202	5.0	55	3.9
**Annual household income before tax**	**5441**					
≤€30,000	1384	25.4	913	22.6	471	33.5
€30,001–€50,000	1376	25.3	1010	25.0	366	26.0
€50,001–€70,000	961	17.7	707	17.5	254	18.1
>€70,000	1720	31.6	1406	34.8	314	22.3
**Use of summer cottage**	**5441**					
0–13 days	3573	65.7	2739	67.9	834	59.4
14 days–2 months	1402	25.8	1026	25.4	376	26.8
>2 months	466	8.6	271	6.7	195	13.9
**Area-level mean income per €1000**	**5441**	27.2	27.2 ^c^	5.7 ^d^	27.2 ^c^	5.8 ^d^
**Area-level unemployment rate (%) ^c^**	**5441**	6.0 ^c^	6.0	1.6 ^d^	6.2	1.6 ^d^
**Wood-burning frequency**	**5441**					
never	4752	87.5	3523	87.3	1229	87.5
1–2 time/week	350	6.4	256	6.3	94	6.7
>2 time/week	339	6.2	257	6.4	82	5.8
**% Coverage of green space within 1 km**	**5441**	32.1 ^c^	32.2 ^c^	15.0 ^d^	32.0 ^c^	14.1 ^d^
**% Coverage of blue space**	**5441**					
0–1	2968	54.5	2197	54.4	771	54.9
1.01–4	1267	23.3	937	23.2	330	23.5
>4	1206	22.2	902	22.3	304	21.6

^a^ For each variable, ‘total N’ denotes the number of respondents within each response category of the variable. ^b^ For each variable, ‘n’ denotes the distribution of response categories within strata of the outcome variable (users or non-users of antihypertensives). ^c^ Numbers presented here represent means. ^d^ Numbers presented here represent the standard deviations.

**Table 2:** Summary values and Spearman’s correlation matrix for urban environmental exposures.

Exposures	Mean (SD)	Minimum	Percentile			Maximum	Wood Smoke PM_2.5_	Road-Traffic PM_2.5_	Road-Traffic Noise	Coverage of Green within 1 km	Coverage of Blue Space
			25th	50th	75th						
Wood smoke PM_2.5_ [µg/m^3^]	0.53 (0.21)	0.07	0.37	0.52	0.67	1.12	1	−0.26 ^a^	0.06 ^a^	0.15 ^a^	−0.43 ^a^
Road-traffic PM_2.5_ [µg/m^3^]	0.58 (0.37)	0.00	0.32	0.49	0.76	2.77		1	0.51 ^a^	−0.3 ^a^	−0.11 ^a^
Road-traffic noise [dB] ^b^	54.04 (54.91)	29.97	51.02	50.02	55.92	75.40			1	−0.12 ^a^	−0.16 ^a^
% coverage of green space within 1 km	32.13 (14.77)	3.27	21.96	30.70	40.67	94.37				1	−0.34 ^a^
% coverage of blue space	4.56 (10.15)	0.00	0.00	0.00	2.63	75.02					1

^a^*p*-value < 0.001. ^b^ Noise summary estimates calculated in Pascals and thereafter converted to Decibels.

**Table 3:** Odds ratios for the associations between environmental exposures and use of antihypertensives in the main model.

Exposures	Crude Model ^a^	Main Model ^b^	Single-Exposure Model ^c^
	OR (95% CI)	OR (95% CI)	OR (95% CI)
Wood-smoke PM_2.5_	1.06 (0.74–1.52)	1.12 (0.78–1.57)	1.10 (0.79–1.54)
Road-traffic PM_2.5_	1.02 (0.79–1.31)	0.97 (0.76–1.26)	0.92 (0.75–1.12)
Road-traffic noise ^d^	0.98 (0.92–1.03)	0.98 (0.93–1.04)	0.98 (0.93–1.03)
% coverage of green space within 1 km ^e^	0.99 (0.94–1.04)	0.99 (0.94–1.04)	0.98 (0.93–1.03)

^a^ Model mutually adjusted for pollutants and green space within 1 km; adjusted also for age and sex. ^b^ Model mutually adjusted for pollutants and green space within 1 km; adjusted also for age, sex, employment status, active smoking, passive smoking, alcohol consumption, BMI, exercise, annual household income, and mean area-level income. ^c^ Model adjusted for age, sex, employment status, active smoking, passive smoking, alcohol consumption, BMI, exercise, annual household income, mean area-level income. ^d^ Estimates for every 5 dB increase in road-traffic noise. ^e^ Estimates for every 10% increase in green space coverage. Odds ratios for wood-smoke PM_2.5_ and road-traffic PM_2.5_ were expressed per 1 µg/m^3^ increase in the concentration of particulate matter.

**Table 4:** Sensitivity models for the associations between environmental exposures and use of antihypertensives.

Exposures	Sensitivity Model-1 ^a^	Sensitivity Model-2 ^b^	Sensitivity Model-3 ^c^	Sensitivity Model-4 ^d^	Sensitivity Model-5 ^e^
	OR (95% CI)	OR (95% CI)	OR (95% CI)		
Wood-smoke PM_2.5_	1.05 (0.69–1.60)	1.14 (0.79–1.65)	1.13 (0.79–1.63)	1.06 (0.73–1.54)	1.15 (0.79–1.66)
Road-traffic PM_2.5_	0.96 (0.74–1.25)	0.97 (0.75–1.25)	0.99 (0.77–1.28)	1.00 (0.77–1.30)	1.03 (0.80–1.33)
Road-traffic noise ^f^	0.97 (0.92–1.03)	0.98 (0.93–1.04)	0.98 (0.93–1.04)	0.97 (0.92–1.03)	0.97 (0.92–1.03)
% Coverage of green space within 1 km ^g^	0.98 (0.93–1.04)	0.99 (0.94–1.05)	-	0.98 (0.93- 1.03)	0.99 (0.94–1.04)
% Coverage of green space within 300 m ^g^			1.01 (0.96–1.06)		
% Coverage of blue space within 1 km					
0–1	1	-	-	-	-
1.01–4	1.06 (0.84–1.33)	-	-	-	-
>4	1.03 (0.80–1.33)	-	-	-	-
Wood burning frequency					
never	-	1	-	-	-
1–2 times/week	-	1.03 (0.77–1.38)	-	-	-
>2 times/week	-	0.85 (0.63–1.15)	-	-	-

^a^ Model mutually adjusted for pollutants and green space within 1 km, and also age, sex, employment status, active smoking, passive smoking, alcohol consumption, exercise, use of a summer home, and access to blue space. ^b^ Model mutually adjusted for pollutants and green space within 1 km, and also age, sex, employment status, active smoking, passive smoking, alcohol consumption, exercise, annual household income, mean area-level income, and frequency of residential wood burning. ^c^ Model mutually adjusted for pollutants and green space within 300 m, and also age, sex, employment status, active smoking, passive smoking, alcohol consumption, exercise, annual household income, and mean area-level income. ^d^ Model mutually adjusted for pollutants and green space within 1 km, and also age, sex, employment status, active smoking, passive smoking, alcohol consumption, exercise, annual household income, mean area-level income and BMI. ^e^ Model mutually adjusted for pollutants and green space within 1 km, and also age, sex, employment status, active smoking, passive smoking, alcohol consumption, exercise, annual household income, area-level unemployment and educational status. ^f^ Estimates for every 5 dB increase in road-traffic noise. ^g^ Estimates for every 10% increase in green space coverage. Odds ratios for wood-smoke PM_2.5_ and road-traffic PM_2.5_ were expressed per 1 µg/m^3^ increase in the concentration of particulate matter.

**Table 5:** Main model odds ratios representing association between environmental exposures and self-reported hypertension (physician diagnosed or treated hypertension).

Exposures	Crude Model ^a^	Main Model ^b^	Single-Exposure Model ^c^
	OR (95% CI)	OR (95% CI)	OR (95% CI)
Wood smoke PM_2.5_	0.82 (0.58 1.16)	0.84 (0.59 1.19)	0.84 (0.61 1.17)
Road-traffic PM_2.5_	1.04 (0.82 1.32)	0.99 (0.78 1.27)	1.00 (0.82 1.21)
Road-traffic noise ^d^	0.98 (0.93 1.04)	0.99 (0.94 1.04)	0.99 (0.94 1.03)
% Coverage of green space within 1 km ^e^	0.98 (0.93 1.03)	0.99 (0.94 1.04)	0.98 (0.93 1.02)

^a^ Model mutually adjusted for pollutants and green space within 1 km, and also age and sex. ^b^ Model mutually adjusted for pollutants and green space within 1 km, and also age, sex, employment status, active smoking, passive smoking, alcohol consumption, exercise, annual household income, mean area-level income. ^c^ Model adjusted for age, sex, employment status, active smoking, passive smoking, alcohol consumption, exercise, annual household income, mean area-level income. ^d^ Estimates for every 5 dB increase in road-traffic noise. ^e^ Estimates for every 10% increase in green space coverage. Odds ratios for wood-smoke PM_2.5_ and road-traffic PM_2.5_ were expressed per 1 µg/m^3^ increase in the concentration of particulate matter.
